# One-year real-world experience with mavacamten and its physiologic effects on obstructive hypertrophic cardiomyopathy

**DOI:** 10.3389/fcvm.2024.1429230

**Published:** 2024-08-30

**Authors:** Daniel Seung Kim, Emily L. Chu, Emily E. Keamy-Minor, Ishan Dhananjay Paranjpe, Wilson L. Tang, Jack W. O’Sullivan, Yaanik B. Desai, Michael B. Liu, Elise Munsey, Kimberly Hecker, Isabella Cuenco, Beth Kao, Ellen Bacolor, Colleen Bonnett, Andrea Linder, Kathleen Lacar, Nancy Robles, Cindy Lamendola, Allysonne Smith, Joshua W. Knowles, Marco V. Perez, Masataka Kawana, Karim I. Sallam, Chad S. Weldy, Matthew T. Wheeler, Victoria N. Parikh, Heidi Salisbury, Euan A. Ashley, the Stanford Center for Inherited Cardiovascular Disease, Karim I Sallam

**Affiliations:** ^1^ Division of Cardiovascular Medicine, Department of Medicine, Stanford University School of Medicine, Stanford, CA, United States; ^2^ Stanford Center for Inherited Cardiovascular Disease, Stanford University School of Medicine, Stanford, CA, United States; ^3^ Wu Tsai Human Performance Alliance, Stanford University School of Medicine, Stanford, CA, United States; ^4^ Center for Digital Health, Stanford University School of Medicine, Stanford, CA, United States; ^5^ Stanford Cardiovascular Institute, Stanford University School of Medicine, Stanford, CA, United States; ^6^ Stanford Diabetes Research Center, Stanford University School of Medicine, Stanford, CA, United States; ^7^ Department of Genetics, Stanford University School of Medicine, Stanford, CA, United States; ^8^ Department of Biomedical Data Science, Stanford University School of Medicine, Stanford, CA, United States

**Keywords:** mavacamten, MYK-461, hypertrophic cardiomyopathy, cardiac myosin inhibitor, hypertrophic cardiomyopathy with obstruction (oHCM), hypertrophic obstructive cardiomyopathy (HOCM), left ventricular outflow tract obstruction

## Abstract

Mavacamten is a first-in-class cardiac myosin ATPase inhibitor, approved by the United States Food and Drug Administration for the treatment of hypertrophic cardiomyopathy with obstructive physiology (oHCM). Here, we present the real-world use of mavacamten in 50 patients with oHCM at a tertiary care referral center. In both our highlighted case and in our aggregate data, we report significant improvement in wall thickness, mitral regurgitation, left ventricular outflow tract obstruction and New York Heart Association symptom class. Moreover, in our center's experience, neither arrhythmia burden, nor contractility have worsened in the vast majority of patients: we note a clinically insignificant mean decrease in left ventricular ejection fraction (LVEF), with only two patients requiring temporary mavacamten discontinuance for LVEF < 50%. Adverse events were rare, unrelated to mavacamten itself, and seen solely in patients with disease too advanced to have been represented in clinical trials. Moreover, our multidisciplinary pathway enabled us to provide a large number of patients with a novel closely-monitored therapeutic within just a few months of commercial availability. These data lead us to conclude that mavacamten, as a first-in-class cardiac myosin inhibitor, is safe and efficacious in real-world settings.

## Introduction

Hypertrophic cardiomyopathy is a genetic disease associated with variants in the genes of the cardiac sarcomere and characterized by enhanced cardiac actin-myosin crossbridge cycling leading to hyperdynamic contractility, cardiac hypertrophy, and diastolic dysfunction (1). Obstructive hypertrophic cardiomyopathy (oHCM) occurs when hypercontractility, hypertrophy, and mitral valve morphology contribute to outflow tract obstruction (1). Mavacamten is a first-in-class, small molecule inhibitor of cardiac myosin ATPase approved by the US Food and Drug Administration (FDA) in April 2022 for use in patients with symptomatic oHCM. Mavacamten reduces contractile force through decreased myosin head availability (2). We now have more than 18 months of experience with commercial mavacamten following the Risk Evaluation and Mitigation Strategies (REMS) pathway, and there are few published reports of its longer term, real-world effects (3). Here, we report a case series of 50 patients with oHCM treated with mavacamten and closely monitored for an average of 36 weeks through a multidisciplinary program at a single center.

## Methods

This was a retrospective observational study of the first 50 adult (age > 18 years old) patients who were evaluated at Stanford University and initiated on mavacamten for symptomatic oHCM. All patients provided informed, written consent and were enrolled in the FDA-mandated Risk Evaluation and Mitigation Strategies (REMS) pathway (4). Electronic medical records were reviewed for baseline and post-mavacamten clinical and echocardiographic data. Appropriate institutional review board approval was obtained from Stanford University.

### Echocardiography

Prior to initiation of mavacamten, all patients underwent comprehensive echocardiogram evaluation to evaluate left ventricular ejection fraction (LVEF), left ventricular outflow tract (LVOT) obstruction gradient, mitral regurgitation severity, maximal thickness of the interventricular septum, right ventricular systolic pressure (RVSP) estimates, and measures of diastolic function. Of note, only the LVEF and LVOT measurements were required for initiation of mavacamten. Per the REMS pathway, patients had follow-up echocardiograms performed at 4, 8, and 12 weeks following mavacamten initiation, with subsequent follow-up every 3 months afterward (4). As echocardiograms could be performed locally due to a patients’ preference, not all the aforementioned measurements were obtained at follow-up. Each echocardiogram was reviewed by the prescribing Stanford physician to confirm LVEF and LVOT gradient measurements prior to the next dispensing of mavacamten.

### Outcomes

Manual chart review and data extraction was performed to obtain baseline and follow-up clinical data. New York Heart Association (NYHA) symptom class was recorded at 4, 8, 12 weeks and at latest follow-up. Adverse events, such as death, hospitalization, temporary/permanent discontinuation of mavacamten, and arrhythmia development (both atrial fibrillation and ventricular tachycardia) were recorded.

### Statistical analysis

All analyses were performed in R (https://www.r-project.org). Continuous variables are presented as mean and standard deviation. Categorical variables are presented as number and percentage. As each patient served as their own pre- and post- comparison, a paired *t*-test was used to test for significant difference across the cohort for continuous variables. Similarly, a McNemar-Bowker test was used to test for significant differences across categorical variables. *P* < 0.05 was considered significant.

## Results

### Case report

A 26-year-old man presented with severely reduced exercise capacity (VO2_max_ 67% of predicted) due to obstructive HCM characterized by severe septal hypertrophy (2.8 cm) with associated late gadolinium enhancement on cardiac magnetic resonance imaging. He had normal-to-hyperdynamic left ventricular ejection fraction (LVEF, 63%) and a resting LVOT gradient of 90 mmHg with systolic anterior motion (SAM) of the mitral valve causing moderate mitral regurgitation. Genetic testing was unavailable in the proband due to lack of insurance coverage, but his sister (who was also seen in our clinic) underwent testing that revealed a likely pathogenic variant in *MYH7* (c.1051A>G, p.Lys351Glu).

**Figure 1:**
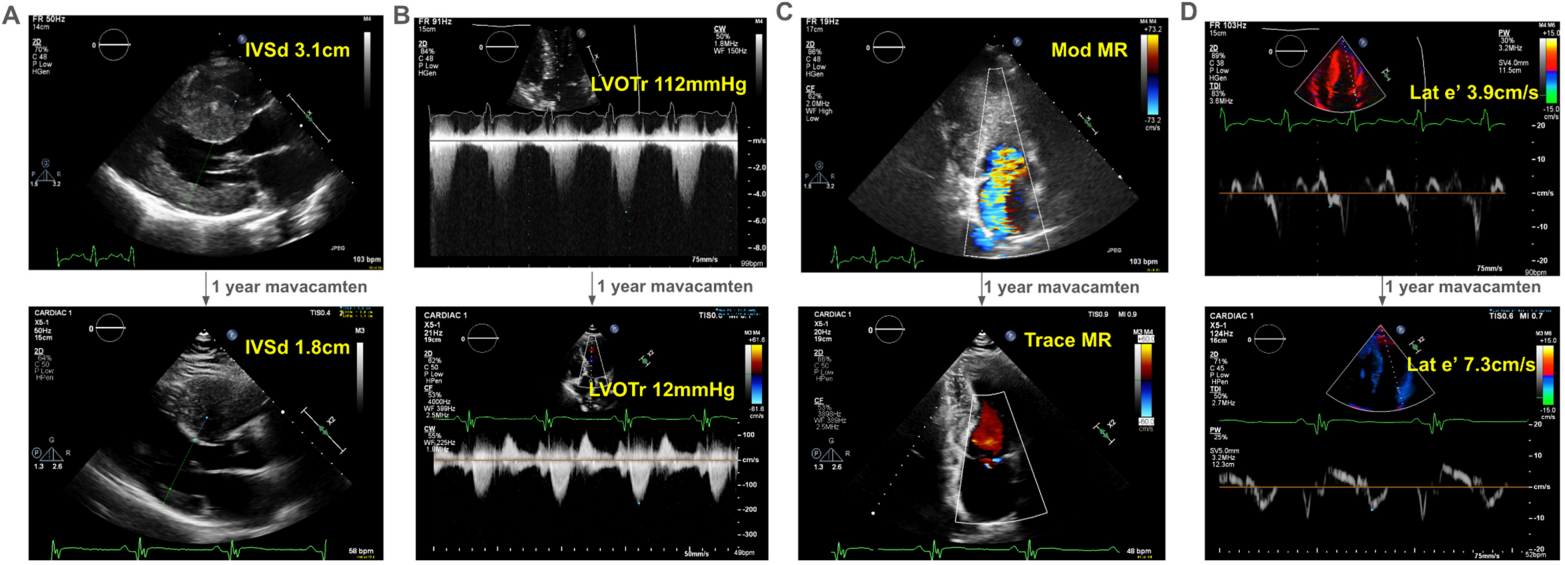
Echocardiographic changes after one-year of mavacamten in a 41-year old man with obstructive hypertrophic cardiomyopathy. With one-year of treatment with mavacamten, our proband experienced improved symptoms, which correlated echocardiographically (see Supplementary Video S1) to **(A)** a decrease in interventricular septum thickness on parasternal long axis view, **(B)** his left ventricular outflow tract gradient at rest, **(C)** the degree of mitral regurgitation, and **(D)** his diastolic function as represented by his lateral e’. IVSd, interventricular septum thickness in diastole in cm; LVOTr, left ventricular outflow tract gradient at rest in mmHg; MR, mitral regurgitation; lat e’, lateral e’ in cm/s, a spectral Doppler measurement reflecting diastolic function.

Over years of follow-up, the patient developed decreasing exercise tolerance, with increasing left ventricular septal hypertrophy to 3.1 cm (Figure 1A) and LVOT gradient of 112 mmHg at rest, with possible left ventricular mid-cavitary gradient that was not formally measured at the time of imaging (Figure 1B). His SAM-associated moderate mitral regurgitation worsened (Figure 1C). He had echocardiographic evidence of diastolic dysfunction, with reduced lateral e’ of 3.9 cm/s (Figure 1D). He deferred surgical myectomy out of concern for operative risk.

He started mavacamten at 40 years of age. After one year, there was dramatic improvement in his symptoms with a reduction in his interventricular septal thickness from 3.1 cm to 1.8 cm (Figure 1A) and resting LVOT gradient from 112 mmHg to 12 mmHg (Figure 1B). Mitral regurgitation reduced to trace (Figure 1C). Finally, he had improvement in his lateral e’ velocity from 3.9 cm/s to 7.3 cm/s (Figure 1D, Supplementary Video S1).

### One year experience of real world mavacamten therapy

We have observed similar, though typically less dramatic trends, across patients on mavacamten. In our cohort, 64% (32) were women with an average age of 63.5 ± 13.5 years (standard deviation, SD) and body-mass index of 28.5 ± 5.4 kg/m^2^. 41 patients (82%) were on either beta-blocker or calcium-channel blocker therapies, with 68% (*N* = 34), 16% (8), and 2% (1) on beta-blocker therapy, calcium channel blocker therapy, or both medication classes, respectively. All patients were closely monitored according to the FDA-mandated REMS pathway (4), with an average 36 weeks follow up. Mavacamten was temporarily held in 5 patients (10%) due to Valsalva LVOT gradient <20 mmHg, and in 2 patients (4%) for LVEF <50%, in both cases for 4-weeks before re-initiation of mavacamten therapy at a lower dose than prior^4^. We note that holding mavacamten for an LVOT gradient <20 mmHg is mandated by the FDA REMS pathway algorithm^4^, despite such a reduction being the desired physiologic effect. Mavacamten was stopped in 3 patients (6%) for fatigue/malaise (*n* = 2), and loss of insurance coverage (*n* = 1). We noted minimal atrial fibrillation or non-sustained ventricular tachycardia on follow-up monitoring. Mavacamten doses on most recent follow-up, adjusted per the FDA REMS pathway algorithm^4^, were: 2.5 mg (9, 20%), 5 mg (17, 38%), 10 mg (13, 29%), and 15 mg (6, 13%, Table 1).

**Table 1:** Baseline characteristics, clinical outcomes, and changes to echocardiographic data with mavacamten treatment in 50 patients with obstructive hypertrophic cardiomyopathy.

Baseline characteristics			
	*N*	Mean (±SD) or % (*N*)	*P*
Age, years	50	63.5 (**±**13.5)	–
Gender,% Female	50	64.0% (32)	–
Body mass index, kg/m^2^	50	28.5 (**±**5.4)	–
Beta-blocker or calcium channel blocker therapy	50	82.0% (41)	–
Beta-blocker therapy	50	68.0% (34)	–
Calcium-channel blocker therapy	50	16.0% (8)	–
Both medication classes	50	2.0% (1)	–
Clinical outcomes with mavacamten treatment			
	*N*	Mean (±SD) or % (*N*)	*P*
Time in Mavacamten REMS, weeks	45	35.9 (**±**17.5)	–
Mavacamten temporarily held,%	50	10.0% (5)	–
Mavacamten stopped,%	50	6.0% (3)	–
New atrial fibrillation,%	50	4.0% (2)	–
New ventricular tachycardia,%	50	2.0% (1)	–
Death, MCS, or transplant,%	50	6.0% (3)	–
	Baseline, % (*N*)	Post-mavacamten, % (*N*)	*P* *
NYHA symptom classification	49	43	<0.001
I - no limitations of activity	0% (0)	44.2% (19)	
II - slight limitations of activity	30.6% (15)	51.2% (22)	
III - marked limitations of activity	69.4% (34)	4.6% (2)	
IV - symptoms at rest	0% (0)	0% (0)	
Mavacamten dose at follow-up	47	45	–
2.5 mg	–	20.0% (9)	–
5 mg	–	37.8% (17)	–
10 mg	–	28.9% (13)	–
15 mg	–	13.3% (6)	–
Change in echocardiographic data with mavacamten treatment			
	*N*	Mean absolute delta (95% CI)*	*P* **
LVOTr, mmHg	37	−33.1 (−45.3, −21.0)	<0.001
LVOTv, mmHg	32	−50.1 (−68.5, −31.7)	<0.001
LVEF	42	−3.1 (−5.4, −0.83)	0.0088
LV interventricular septum, cm	42	−0.22 (−0.30, −0.13)	<0.001
E/e’	19	−4.0 (−9.7, 1.7)	0.15
Lateral e’, cm/s	18	−0.53 (−1.9, 0.87)	0.43
Medial e’, cm/s	21	−0.88 (−1.7, −0.087)	0.032
RVSP, mmHg	23	−2.5 (−5.6, 0.67)	0.12

	Baseline, % (*N*)	Post-mavacamten,% (*N*)	*P* *
MR Severity	47	39	<0.001
None/Trace	14.9% (7)	33.3% (13)	
Mild	21.3% (10)	51.3% (20)	
Moderate	44.7% (21)	12.8% (5)	
Moderately-Severe	10.6% (5)	2.6% (1)	
Severe	8.5% (4)	0% (0)	

LV, left ventricle; LVEF, left ventricular ejection fraction; LVOTv, left ventricular outflow tract obstructive gradient with Valsalva maneuver; LVOTr, left ventricular outflow tract obstructive gradient at rest; MCS, mechanical circulatory support; MR, mitral regurgitation; NYHA, New York Heart Association; RVSP, right ventricular systolic pressure; SD, standard deviation.

* McNemar-Bowker test used for calculation of *P*-value.

** Paired sample *t*-test used for calculation of *P*-value and 95% confidence intervals.

We observed significant changes in echocardiographic measurements of mavacamten-treated patients at most recent follow-up compared to baseline. In line with trial data, there was a dramatic mean decrease in LVOT gradient at rest and with Valsalva with reductions of −33 mmHg [−45, −21] (95% CI) in resting LVOT gradient, and −51 mmHg [−69, −32] reduction in Valsalva LVOT gradient (*P* < 0.001, pairwise *T*-test.) There was significant improvement in the proportion of patients with moderate or severe mitral regurgitation (*P* < 0.001, McNemar-Bowker, Table 1). Accompanying this change in LVOT gradient, we noted a significant decrease in diastolic interventricular septal thickness [−0.2 (−0.30, −0.13) cm, *P* < 0.001, Table 1]. We observed a clinically insignificant reduction in mean LVEF from 67% to 64% [−3.1 (−5.4, −0.8)%, *P* = 0.008] without change in right ventricular systolic pressure (*P* = 0.12, Table 1). There was a significant decrease in medial e’ [−0.9 cm/s (−1.7, −0.09), *P* = 0.03], but no significant change in E/e’ or lateral e’ (Table 1). We note that the changes in measures diastolic function may reflect mavacamten-specific improvements in diastolic stiffness likely owing to stabilization of the super-relaxed state of myosin (5). These positive changes in echocardiographic measurements were mirrored by New York Heart Association (NYHA) symptom class, which improved dramatically with mavacamten treatment (McNemar-Bowker test, *P* < 0.001).

To compare the findings of our patients who started on mavacamten, we considered a subset of patients (*N* = 5) with symptomatic oHCM (NYHA symptom classification ≥ II), referred for mavacamten but who ultimately did not start the medication (Table 2). These patients had an average age of 70.6 years, 60% (3) were female, had an average BMI of 32.2, and 80% of the participants (4) on beta-blockers; overall similar in demographics and baseline clinical characteristics to the subset who received mavacamten. In contrast to those started on mavacamten, there was slight worsening in NYHA symptom classification in follow-up without mavacamten, with two patients previously NYHA II progressing to NYHA III symptoms. In addition, LVOT gradients increased by 19.2 mmHg and 13.2 mmHg during the follow-up period for gradients at rest and with Valsalva. These physiologic changes were accompanied by an increase in E/e’ of 2.9 and slight decreases of 0.6 cm/s and 0.8 cm/s in lateral and medial e’, respectively. There were small changes in LVEF (+2%) and interventricular septum thickness (−0.04 cm). One patient improved their mitral regurgitation classification from moderate to mild.

**Table 2:** Clinical characteristics of a subset of patients referred for mavacamten, who ultimately did not start the medication.

Baseline characteristics		
	*N*	Mean (±SD) or % (*N*)
Age, years	5	70.6 (**±**6.4)
Gender,% Female	5	60% (3)
Body mass index, kg/m^2^	5	32.2 (**±**3.52)
Beta-blocker or calcium channel blocker therapy	5	80% (4)
Beta-blocker therapy	5	0
Calcium-channel blocker therapy	5	0
Both medication classes	5	0
Changes since referral for mavacamten		
	Baseline,% (*N*)	Post-mavacamten referral,% (*N*)
NYHA symptom classification	5	5
I - no limitations of activity	0	0
II - slight limitations of activity	4	2
III - marked limitations of activity	1	3
IV - symptoms at rest	0	0
Change in echocardiographic data with mavacamten treatment		
	*N*	Mean absolute delta
LVOTr, mmHg	5	+19.2
LVOTv, mmHg	5	+13.2
LVEF	5	+2
LV interventricular septum, cm	5	−0.04
E/e’	2	+2.9
Lateral e’, cm/s	2	−0.6
Medial e’, cm/s	2	−0.8
RVSP, mmHg	1	−14.0
	Baseline,% (*N*)	Post-mavacamten referral,% (*N*)
MR severity	5	5
None/Trace	20% (1)	40% (2)
Mild	60% (3)	40% (2)
Moderate	20% (1)	20% (1)
Moderately-severe	0	0
Severe	0	0

LV, left ventricle; LVEF, left ventricular ejection fraction; LVOTv, left ventricular outflow tract obstructive gradient with Valsalva maneuver; LVOTr, left ventricular outflow tract obstructive gradient at rest; MR, mitral regurgitation; NYHA, New York Heart Association; RVSP, right ventricular systolic pressure; SD, standard deviation.

Two elderly patients with severe concomitant disease unrelated to oHCM (4%) died during follow up: One had pre-existing hypoxemic pulmonary hypertension that progressed. The second died from unrelated septic shock. One patient (2%) developed septic shock due to tricuspid valve endocarditis during which mavacamten was discontinued, and ultimately required ECMO and complex cardiac surgery. Only a limited subset of our cohort had pre- and post-mavacamten cardiopulmonary exercise stress testing (*n* = 7), precluding meaningful statistical analysis.

To enable timely and optimally monitored access to cardiac myosin inhibitor therapy for our patients, our center developed a nurse-led training course for the FDA REMS program (4). This training focuses on appropriate patient selection, prescription, authorization, triage and surveillance, and provides education on common clinical problems that can arise during therapy with an emphasis on interdisciplinary care. Particular emphasis was placed on REMS monitoring and dose adjustment based on echocardiographic parameters.

## Discussion

Limitations of our real-world data include the lack of a single center performing all laboratories and echocardiograms – as a result, not all echocardiographic parameters were reported on follow-up imaging performed at local centers (per patient preferences) and an inability to track changes in laboratories (e.g., nt-proBNP and troponin levels) longitudinally. In addition, while we have collected one of the largest single-center collection of patients with oHCM on mavacamten, our sample size of 50 patients may be prone to regional bias of the population of patients served by our center. These limitations combine to potentially limit the generalizability of our findings. As a result of these shortcomings, we are actively collaborating in the HCM SHaRe registry to pool data with other centers to ensure that future analyses are representative of the larger United States population and sufficiently statistically powered to draw inferences from clinical, laboratory, and echocardiographic data (6).

Our novel data is one of the first real-world reports of mavacamten use in oHCM patients (3) and the resultant physiologic and clinical responses due to cardiac myosin inhibition (7, 8). Specifically, we highlight that mavacamten results in significant improvement in wall thickness, mitral regurgitation, LVOT obstruction (both at rest and with Valsalva), and symptomatic improvement in NYHA class outside of the clinical trial setting. Moreover, we did not find worsened arrhythmia burden or reductions in contractility in the vast majority of patients (two patients required temporary discontinuance for LVEF < 50%, as mandated by the FDA REMS monitoring (4). Adverse events were rare, unrelated to mavacamten itself, and seen solely in patients with disease too advanced to have been represented in clinical trials. These data lead us to conclude that mavacamten, as a first-in-class cardiac myosin inhibitor (7, 8), is safe and efficacious in real-world settings and can be broadly used in clinical practice for treatment of symptomatic oHCM.

## Data Availability

The raw data supporting the conclusions of this article will be made available by the authors, without undue reservation.

## Stanford center for inherited cardiovascular disease author masthead

Karim I Sallam, Masataka Kawana, Chad S Weldy, Marco Perez, Joshua W Knowles, Jason Tso, Cindy Lamendola, Allysonne Smith, Nancy Robles, Colleen Bonnett, Ellen Bacolor, Kimberly Hecker, Isabella Cuenco, Beth Kao, Elise Munsey, Andrea Linder, Kathleen Lacar, Julia Platt, Chloe Reuter, Tia Moscarello, Ryan Murtha, Jennifer Kohler, Hannah Ison, Mitchel Pariani, Anusha Klinder, Priya Nair, Jennifer Marino, Andrea Linder, Ruchi Patel, Matthew T Wheeler, Euan A Ashley, Victoria N Parikh.
